# The complete mitogenome of *Nomia chalybeata* (Hymenoptera: Halictidae) and phylogenetic analysis

**DOI:** 10.1080/23802359.2020.1790320

**Published:** 2020-07-15

**Authors:** Hongying Li, Huanhuan Lu, Shigui Huang, Xiaodong Fan, Ancai Luo, Dunyuan Huang

**Affiliations:** a Chongqing Key Laboratory of Vector Insects, College of Life Sciences, Chongqing Normal University, Chongqing, PR China; b The Qiyunshan National Nature Reserve, Jiangxi, PR China

**Keywords:** *Nomia chalybeata*, Hymenoptera, mitogenome, phylogeny

## Abstract

The complete mitogenome of *Nomia chalybeata* was sequenced. The mitochondrial length of *N. chalybeata* was 16,692 bp (AT content 85.4%), with 37 classic invertebrate mitochondrial genes (including 13 protein-coding genes, 22 transporter RNAs, and two ribosomal RNAs) and AT-rich region (AT content 91.7%). The maximum-likelihood phylogenetic relationship was constructed using 11 species from Hymenoptera. Through the phylogenetic relationship, our research team successfully used the molecular data of the mitochondrial genome to verify that *N. chalybeata* belongs to the family Halictidae, and also provides molecular data for the database of the family Halictidae.

*Nomia chalybeata* belongs to the subfamily Nomiinae of the family Halictidae within the Hymenoptera. In 1875, the species was officially named by Smith (1875). At present, among the subfamily Nomiinae, about 520 species of 11 genera are known in the world, and 42 species of 4 genera are known in China, which records only 8% of world records (Huang 2008). *N. chalybeata* is widely distributed in China, including Sichuan, Jiangxi, Taiwan, and other places, and abroad in Japan, South Korea, Indonesia, India, Myanmar, and other countries. Here, our research team sequenced the mitogenome of *N. chalybeata*, which was collected in Xining area, 108 National Road, Xichang City, Liangshan Yizu Autonomous Prefecture, Sichuan Province, China (N 27.738288, E 102.233128). The samples used in the experiment are now stored in the Chongqing Key Laboratory of Vector Insects, Chongqing Normal University (Accession number: LHY-2019-LCDF-1) and the GenBank accession No. is MT645078.

The full-length mitochondrial sequence of *N. chalybeata* is 16,692 bp, of which AT content is 85.4%. The mitochondria have 37 structures (13 PCGs, 22 tRNAs, and two rRNAs) that are common in invertebrates. The AT-rich region is also located in this mitogenome, the AT content is 91.7%. Compared to other Hymenoptera (Zheng et al. 2018; Lu and Huang 2020), the mitochondria of *N. chalybeata* have similar genetic composition and arrangement.

The start codon of 13 PCGs is ATN (including four ATG, four ATA, and five ATT), which uses 12 TAA and one TAG stop codon to end sequence coding. A total of 22 tRNAs genes have a cloverleaf structure except for *trnS1,* whose dihydrouridine (DHU) arm forms a simple loop and the phenomenon has been reported in other Hymenopteran species (He et al. 2019; Xiong et al. 2019). The 16S rRNA (*rrnL*) and 12S rRNA (*rrnS*) genes are located on the minority stand, at the same time, the two genes are separated by trnV, which is common in the mitogenomes of Hymenoptera (Huang et al. 2016). The AT-rich region was 1044 bp long.

To clarify the position of *N. chalybeata* in the Hymenoptera phylogenetic network, our research team downloaded 13 mitogenome sequences from NCBI for analysis. In addition, *Abispa ephippium* and *Philanthus triangulum* are outgroups. Phylogenetic tree was constructed from the 13 PCGs using maximum-likelihood (ML) phylogenies which was inferred using IQ-TREE version 1.6.8 (Nguyen et al. 2015).

Each node of the phylogenetic tree has a high degree of credibility (Figure 1). The phylogenetic network formed by the species selected in this study is consistent with previous studies (He et al. 2018). It can be seen from the phylogenetic tree that *N. chalybeata* converges with Genus Lasioglossum bees (*Lasioglossum lativentre and Lasioglossum sp. SJW 2017*) to be supported, this shows that at the molecular level, Nomia Chalybeata belongs to the family Halictidae. In addition, *N. chalybeata* is more closely related to Colletidae than to Apidae and Megahilidae. When our research team search the NCBI for the complete mitochondrial genome of the family Halictidae species, only three genome sequences are available. Therefore, the mitochondrial genome can effectively supplement the mitochondrial database of the family Halictidae.

**Figure 1.**
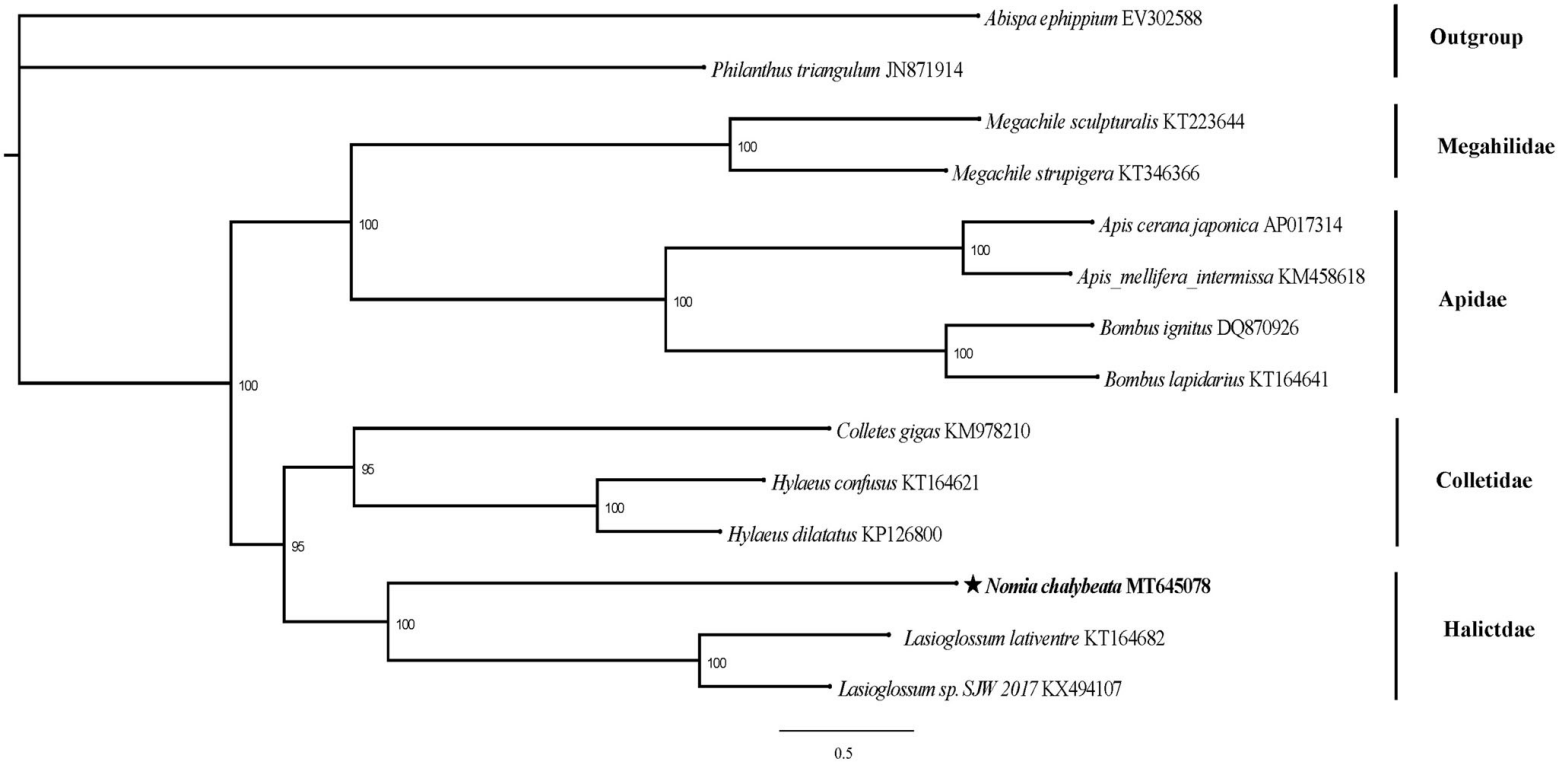
Phylogenetic relationships of *Nomia chalybeata* were constructed using PCGs sequences from 13 species. GeneBank No. is displayed behind each species name.

## Data Availability

The mitochondrial genome data used in this study can be obtained free of charge on NCBI through GeneBank No. (NCBI https://www.ncbi.nlm.nih.gov/).
